# Circular RNA: A promising new star of vaccine

**DOI:** 10.2478/jtim-2023-0122

**Published:** 2023-12-20

**Authors:** Jindong Xie, Fengxi Ye, Xinpei Deng, Yuhui Tang, Jie-Ying Liang, Xufeng Huang, Yuying Sun, Hailin Tang, Jinsong Lei, Shaoquan Zheng, Yutian Zou

**Affiliations:** State Key Laboratory of Oncology in South China, Guangdong Provincial Clinical Research Center for Cancer, Sun Yat-sen University Cancer Center, Guangzhou 510060, Guangdong Province, China; Zhongshan School of Medicine, Sun Yat-sen University, Guangzhou 510060, Guangdong Province, China; Guangdong Provincial Key Laboratory of Malignant Tumor Epigenetics and Gene Regulation, Department of Medical Oncology, Sun Yat-sen Memorial Hospital, Sun Yat-sen University, Guangzhou 510000, Guangdong Province, China; Department of Data Science and Visualization, Faculty of Informatics, University of Debrecen, Debrecen, Hungary; Breast Disease Center, The First Affiliated Hospital of Sun Yat-sen University, Guangzhou 510000, Guangdong Province, China

**Keywords:** circular RNA, vaccine, drug delivery, immunotherapy

## Abstract

Circular RNAs (circRNAs) are a class of single-stranded RNAs with covalently closed structures. Owing to their not having 3' or 5' ends, circRNAs are highly durable and insusceptible to exonuclease-mediated degradation. Moreover, some circRNAs with certain structures are translatable, making them novel vaccines. Vaccines are efficient tools for immunotherapy, such as for the prevention of infectious diseases and cancer treatment. The immune system is activated during immunotherapy to fight against abnormal allies or invaders. CircRNA vaccines represent a potential new avenue in the vaccine era. Recently, several circRNA vaccines have been synthesized and tested *in vitro* and *in vivo*. Our review briefly introduces the current understanding of the biology and function of translatable circRNAs, molecular biology, synthetic methods, delivery of circRNA, and current circRNA vaccines. We also discussed the challenges and future directions in the field by summarizing the developments in circRNA vaccines in the past few years.

## Introduction

Vaccines are essential for the maintenance of human health. They are the most efficient tools for infectious disease prevention and among the most promising methods for fighting cancer. The vaccination era began in the late 18th century when Edward Jenner invented the first vaccine to induce preventive immunity in the human body to protect against deadly smallpox.^[1]^ Since then, vaccine strategies have evolved from live pathogen inactivation or attenuation techniques to the construction of biologically modified proteins or peptide antigens and eventually to the use of nucleic acids to induce *in vivo* antigen-encoding.^[2,3]^ Although advancements in conventional vaccine approaches have had success in controlling infectious diseases and cancer therapy, challenges remain, and breakthroughs in vaccine technologies are necessary.^[4]^ For example, mRNA vaccines, such as BNT162B2^[5,6]^ and Chadox1^[7]^ have reduced the hospitalization rates of patients infected with the SARS-Cov-2 alpha-variant, but their effectiveness in delta-variant patients is considerably lower than that.^[8, 9, 10]^ Moreover, the protein-encoding functions of circRNAs were demonstrated, and a new type of vaccine using circRNAs as a platform was developed and proved efficient *in vitro* and *in vivo*. These single-stranded RNAs are perfect vaccine carriers because they are highly durable and invulnerable to exonuclease owing to the inaccessibility of free 5’ or 3’ ends.^[11]^ Moreover, they have several advantages that facilitate their use as cost-effective vaccines.

## Overview of circular RNA

### Structure and biogenesis

Circular RNA is a prominent feature of the transcriptomes of many metazoans, including those in humans.^[12, 13, 14, 15]^ Once thought to be meaningless by-products of splicing errors,^[16]^ circRNAs are expressed in cell-specific, tissue-specific, and developmental stage-specific patterns.^[17, 18, 19]^ Known as a type of covalently closed single-stranded RNA without accessible 5’ or 3’ends, circRNAs are usually produced by classical linear splicing and RNA back-splicing.^[20, 21, 22]^ Some circRNAs originate from tRNA splicing reactions.^[23]^ For example, tric31905 is a circular RNA transcript derived from the fruit fly tRNA gene CR31905^[23]^ and the circular tRNA^Trp^ intron is a highly stable circRNA in *Haloferax volcanii*.^[24]^ Linear splicing generally forms intronic circRNAs, upon which an intron lariat that can avoid debranching is created when an upstream donor splice site is ligated to a downstream acceptor splice site.^[20]^ Back-splicing is a process through which a 3′, 5′-phosphodiester bond is formed between the 3′-end of an exon and the 5′-end of either its or an upstream exon, creating a closed structure with a back-splicing junction site.^[12,25]^ Most circRNAs are exon-containing and are derived from the back-splicing of precursor messenger RNA exons in a spliceosome-dependent manner. However, a small portion of intronic circRNAs originates from the processing of cellular non-coding sequences.^[26]^ For example, circRNAs can be detected as processing intermediates during rRNA maturation in archaea.^[24]^ circRNA biogenesis is modulated by various distinct factors.^[17]^ Studies have revealed that back-splicing requires spliceosomal machinery as a catalyst and is regulated by both RNA-binding proteins and intronic complementary sequences.^[27, 28, 29]^ However, additional regulatory factors that influence circRNA biogenesis require further research.

### Function

circRNAs participate in various biological processes, such as cellular activity modulation, gene and protein sponging, cell proliferation, tumor progression, and immune regulation.^[30, 31, 32, 33, 34, 35, 36, 37]^ For decades, circRNAs have been considered untranslatable because they do not have 5’caps,^[38,39]^ and cap-independent translation was discovered to allow circRNA translation;^[40]^ further investigations have provided evidence for their ubiquity.^[41]^ Based on the previous study, Kozak consensus sequences, m6A-modification, and internal ribosome entry sites (IRES) can initiate protein translation from circRNA without 5′ caps.^[20]^ The traits of circRNAs associated with their coding capability are as follows: (1) the presence of IRES in open reading frames (ORFs), N6-methyladenosine (m6A) RNA modification, or Kozak consensus sequences ^[42]^; (2) ribosome association by ribosome profiling; and (3) translated peptides from back-splicing junction sites.^[43]^ According to the literature, circRNAs with nuclear localization are virtually untranslated, and hundreds of circRNAs localized in the cytoplasm are translatable.^[43,44]^ CircRNAs could be incorporated into the 40S subunit of eukaryotic ribosomes *via* IRES, which are upstream of the circRNA start codon (first identified in picornavirus mRNAs), and are thereby translated by ribosomes.^[45]^ Evidence has shown that either the complementary regions of 18S rRNA or a structured RNA element in circRNAs can facilitate circRNA translation in an IRES-dependent manner.^[46]^ Moreover, by recruiting the m6A reader YTHDF3 and translation initiation factor eIF4G2, m6A modifications can initiate circRNA translation.^[47,48]^ Proteins translated from circRNAs are involved in various physiological functions, including the regulation of cell proliferation, differentiation, migration, and myogenesis.^[49, 50, 51, 52, 53]^ The protein-coding ability of circRNAs enables their potential use as cancer vaccines.

### Characteristics that enable circRNA’s use as vaccines

circRNAs have unique characteristics that enable their use as vaccines. Because circRNAs have a covalently closed structure and no termini, circRNAs are immune to degradation mediated by exonucleases, conserving a higher stability than their linear mRNA isoforms. ^[11,25,54,55]^ In mammalian cells, the intermediate half-life of circRNAs is at least 2.5 times more than that of their linear counterparts.^[56]^ The safe antigen production of circRNA-based vaccines can be ensured by circRNAs’ ability to be expressed endogenously.^[41,57–59]^ circRNAs such as circZNF609,^[53]^ circMbl,^[60]^ and circSfl ^[61]^ are associated with polyribosomes and can generate functional proteins that play a role in cell biology.^[49,51,62]^ Without integrating into the genome, circRNA-based vaccines can be translated into antigens in the cytoplasm. In addition, another study demonstrated that synthetic circRNAs derived from foreign introns triggered effective immune responses and inhibited infection *via* the RNA pattern recognition receptor retinoic-acid-inducible gene-I-dependent pathway.^[63]^ These findings indicate that circRNAs can induce innate immune responses and implies that their immunogenicity could be used in creating circRNA self-adjuvant vaccines.^[63]^ Although literature has widely accepted that the translation efficiency of circRNAs in a CAP-independent manner is relatively lower than that of linear RNAs in a CAP-dependent manner, a recent study proposed a different view: circRNAs have high translation efficiency owing to their covalently closed topology. This unique structure allows ribosomes to pass over the length of circRNAs even after reaching a stop codon, which makes it easier to re-initiate translation.^[64]^ In addition, studies have shown that the rolling circle translation of circRNAs is enabled by their covalently closed topology.^[40,42,65]^ Moreover, as recent studies have demonstrated,^[66,67]^ engineering circRNAs can enhance their translation efficiency and lead to more stable protein production than that of their linear counterparts.

Because of inherent stability, immunogenicity, achievable high translation efficiency through engineering, and an inevitable need for nucleotide modifications (which are required for mRNA production *via* the *in vitro* transcription [IVT] pathway for the improvement of mRNA stability without increasing the likelihood of unwanted immunogenicity^[68]^), we posit that circRNAs have the better potency to be developed into vaccines than mRNA.

## Molecular biology of circRNA vaccine

### Structure of vaccine

The circRNA vaccine is a subtype of the nucleic acid vaccine that can be delivered with or without a carrier; thus, the circRNA can be administered directly in a naked form or encapsulated in delivery carriers, such as lipid nanoparticles (LNPs). Modified circRNAs generally comprise coding regions for vaccine antigens, untranslated regions, promoters, IRES, permuted intron-exon (PIE) systems, RNA spacers, and homology arms. IRES are internal ribosome entry sites placed before the coding sequences to initiate translation. Homology arms and rationally designed spacers are constructed to increase circularization efficiency.^[69]^

### Mechanisms of circRNA vaccine-mediated immunotherapy

CircRNA vaccines are promising candidates for disease-related immunotherapies, such as the prevention of infectious diseases and cancer treatment. CircRNA vaccines can generate corresponding proteins *via* translation after injection. These heterologous gene expression products can directly affect immune cells. They induce and reinforce innate and adaptive immunity by stimulating the activation and proliferation of immune cells, reinforcing the host’s ability to fight viruses and tumors. For example, Yang *et al*. reported that direct administration of a circular mRNA (cmRNA) mixture that encodes four cytokines into tumors can lead to T cell activation (both CD4^+^ and CD 8^+^ T cells) and promote immune cell penetration into the tumor, exerting a strong antitumor effect.^[70]^

There is evidence that the activation of immunostimulatory RNA receptors in cells can intrinsically induce immunogenic circRNAs.^[63]^ Owing to their immunogenicity, circRNAs can serve as self-adjuvanted vaccinations. For example, in studies led by Qu L and colleagues, the CircRNA^RBD^ vaccine triggered distinct Th1-skewed immune responses and brought about neutralizing antibody elicitation in a large proportion of patients,^[71]^ suggesting activated innate immune responses.

### Advantages of circRNA vaccine

The most commonly used vaccines are based on pathogens, DNA, and proteins/peptides, each of which has distinct features and limitations. For example, mRNA-based vaccines have short half-lives and are susceptible to exonuclease digestion. Therefore, they require a strictly controlled sterile environment free of ribonucleases during the entire production procedure and a low-temperature cold chain for storage and distribution.^[72]^ DNA-based vaccines have the potential for genome integration.^[73]^

Owing to their unique properties, circRNA vaccines are better than conventional vaccines. First, because circRNAs can produce more significant amounts of proteins for a longer duration than linear RNAs,^[69]^ circRNA vaccines probably have high translation efficiency. The translated products would have prolonged expression. Second, the literature synthesized circRNAs that, even in modest quantities, expressed particular antigens highly immunogenic for DC presentation.^[74]^ This feature results in circRNAs having potential for use in highly efficient vaccines. Third, circRNA-LNPs exhibited greater thermostability than linear mRNA-LNP vaccines. The literature reported that circular RNA vaccines encapsulated in LNPs were conserved well for no less than 4 weeks at 4°C and approximately 30 d at room temperature.^[20]^

However, the limitations and disadvantages of the circRNA vaccines remain unclear. For example, circRNA immunogenicity can be a double-edged sword. Although the immunogenicity of circRNAs enables highly efficient circRNA vaccines, whether this characteristic would inhibit vaccine development remains unclear.^[75]^ Moreover, the safety concerns of circRNA vaccines require further research. In addition, the techniques for generating circRNA vaccines are immature for large-scale vaccine production. Thus, further investigations are necessary before circRNA vaccines can be used in clinical trials.

## Approaches to synthesize circRNA vaccine

Generally, circRNAs are produced by the synthesis of one or more precursor linear RNAs, followed by RNA circularization to generate a covalently closed loop, which is often mediated by chemical or enzymatic ligation methods.^[76]^

### Designing the constructs/backbone of circRNA and linear RNA

A significant challenge in the synthesis of circRNAs is their inability to progress through the standard cap-dependent procedure used to initiate translation. Several strategies have been developed to overcome this limitation. Two main mechanisms allow for endogenous circRNA translation in a cap-independent manner: m6A alteration of the region located close upstream of the start codon and a virally originated IRES upstream of the circular sequence containing the ORF. m6A modification refers to the methylation of N6 in the nitrogenous base adenine,^[77]^ which is the most pervasive modification in eukaryotes.^[78]^ IRES, a structured RNA element located upstream of the start codon, can initiate translation without the presence of a 5′ cap by recruiting eukaryotic ribosomes.^[65]^ The m6A modification is preferable to viral IRESs when considering construct size.^[79]^ However, the two strategies can be combined. For example, m6A increases the effectiveness of circZNF609 translation mediated by IRES. ^[53,80]^ Moreover, a recent study improved circRNA translation. According to Chen et al., the optimization of these five elements—5’ and 3’ UTRs, vector topology, synthetic aptamers, and IRESs—can not only lead to an increase in protein yields of circRNA by several hundred folds but also enable powerful, long-lasting protein production *in vivo*.^[66]^

### Synthesis of precursor linear RNA

There are two methods for synthesizing the precursor linear RNAs for circRNA synthesis: chemical and enzymatic.^[81,82]^ Techniques for RNA chemical synthesis depend on synthesizer machines. Phosphoramidites, derivatives of nucleotide triphosphates, are used in the procedure as fundamental components of linear oligonucleotides.^[79]^ Phage RNA polymerases are used in enzymatic strategies for IVT. An IVT reaction is usually conducted using the following main components: a double-stranded DNA template, ribonucleotide triphosphates, and DNA-dependent RNA polymerase.^[79]^

### RNA circularization in vitro

Generally, exogenous RNA circularization can be achieved using three strategies: chemically, using cyanogen bromide or a similar condensing agent; enzymatically, using DNA or RNA ligases; or ribozymatically, using self-splicing introns.^[69]^

### Chemical strategies

The most commonly used approach is applying 1-Ethyl-3-(3-dimethylaminopropyl) carbodiimide or the condensation reagent cyanogen bromide to activate RNA circularization.^[83]^ Additional chemical methods rely on replacing native phosphate and hydroxyl groups of linear RNA with other functional groups. These alternative reactive groups help form bonds with excellent selectivity and efficacy, increasing the purity and quantities of circularized RNA.^[84]^

### Enzymatic strategies

Three types of polynucleotide ligases are commonly used in the enzymatic ligation of synthetic oligonucleotides: T4 RNA ligase 1, T4 DNA ligase, and T4 RNA ligase 2. They are encoded in the genome of the bacteriophage T4 and can ligate nicks in single-and/or double-stranded RNA constructs. ^[85,86]^ They assist in the circularization process by promoting the ATP-consuming synthesis of a phosphodiester bond between the 3′-hydroxyl (acceptor) and 5′-phosphate (donor) end groups in RNA or DNA.^[87]^

### Ribozymatic strategies

Ribozymatic strategies are the most commonly used methods for RNA circularization, particularly when the production of large circular RNAs is required.^[88]^ Group I and group II introns play a role in circRNA production *in vitro*.^[89, 90, 91]^ However, the use of group I introns is more common than that of group II. In 1992, Puttaraju and Been reported the first use of the group I intron system to synthesize circRNAs from a linear precursor containing introns. The Anabaena pre-tRNA^Leu^ gene was used to form a PIE construct, in which the 5′half of the group I tRNA intron was moved to the tail of the tRNA exon, the remaining 3′half of the intron was placed at the head of the same exon, and circRNAs were formed through exon ligation.^[92]^ The *in vitro* circularization of RNAs can be achieved more efficiently than by using the aforementioned method by using group II introns and does not require native exons.^[89]^ In this pathway, the circularized exon and the excised group II intron are joined together by 3′-5′ and 2′-5′ phosphodiester linkages separately. However, the exact mechanism underlying this process remains unclear.^[93]^

## Delivery of circRNA vaccine

The goal of a vaccine is not only to elicit an initial response to specific antigens but also to promote an enduring response from the body’s adaptive immune system.^[94]^ By exposing the immune system to a particular antigen (or antigens), a vaccine can generate long-lived protective immunity in the form of memory T cells, memory B cells, and antibody-producing plasma cells.^[95]^ Appropriate vaccine delivery is vital to guarantee successful vaccination.

CircRNAs function by producing specific antigens in the host’s cytoplasm. However, they are too large to diffuse freely across cell membranes. CircRNAs might benefit from RNA delivery mechanisms already present, and their distinct structural features might help with formulation and distribution. The terminus-free structure of circRNAs enables resistance to exonuclease degradation, making them suitable for carrier-free administration.^[96]^ For example, naked cmRNA dissolved in PBS was directly injected into the tumor tissues of four types of tumor models, resulting in the detection of its specific expression product.^[70]^ The literature demonstrated that conventional physical techniques such as gene guns, electroporation, and microneedles improved the efficacy of naked mRNA antigen presentation.^[97]^ These results imply that the same methods can be used to facilitate the administration of naked circRNA vaccines. CircRNAs can be delivered to carriers such as nanoparticles. Among all mRNA delivery vehicles, the lipid-based nanoparticle delivery system is the most efficient and occupies the leading status.^[98]^ Its advantages include biocompatibility, ease of formation, modularity, and substantial payload capacity.^[99]^ Moreover, the relatively compact structure of circRNAs may contribute to their high loading capacity into RNA delivery carriers such as viral carriers or nanoparticles.^[20]^ In improving their efficiency, circRNAs are often administered *via* nanocarriers. For instance, purified hEpo circRNAs encased in LNPs demonstrated robust expression when injected into 293 cells, demonstrating the potency of *in vivo* circRNA delivery *via* lipid nanoparticles.^[100]^ Although researchers have managed to deliver functional circRNAs in naked and carrier-facilitated forms, the use of RNA delivery carriers, especially nanoparticles, is a more commonly used method for vaccine delivery.

**Figure 1:**
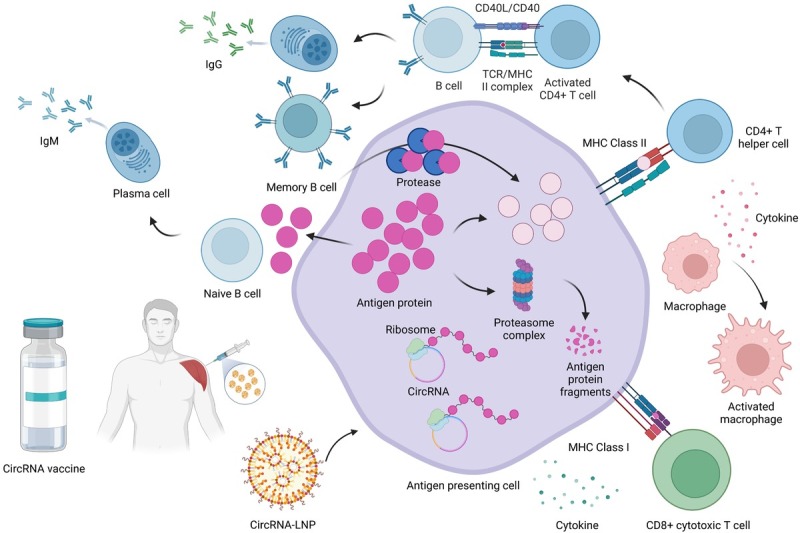
CircRNA *in vivo* delivery and immunity activation. A circRNA vaccine is administered *via* the intramuscular route into the deltoid. circRNA-LNPs are taken up by an APC (antigen-presenting cell) through fusion with the endosomal membrane. CircRNAs combine with ribosomes and are translated into antigen proteins. The intracellular antigen is broken down into smaller fragments by the proteasome complex, and the fragments are displayed on the cell surface to cytotoxic T cells (CD8^+^ T cells) via attachment to MHC I (MHC I is expressed on the surface of nucleated bodily cells). Cytotoxic T cells (CD8^+^ T cells) become activated through recognition and binding to the epitope-bound MHC I receptor, secreting cytokines and cytolytic molecules and thus initiating the cellular immune response. Translated antigen proteins can be secreted outside the cell. These secreted antigens can activate a B cell through BCR (B cell receptor) and make it transform into a plasma cell that secretes IgM antibodies. They can also be taken up by APCs, degraded inside endosomes, and presented on the cell surface by MHC class II proteins. When a CD4^+^ T cell interacts with the APC, it becomes activated and facilitates the activation of macrophages and somatic hypermutation within B cells. These B cells can either be stored as memory cells or disseminate into the blood as plasma cells secreting IgG antibodies.

Immune reactivity after vaccine injection is a key factor affecting vaccine function. The immune response begins as soon as the circRNA reaches the cytoplasm. In this study, we used an LNP-encapsulated circRNA vaccine. After the injection of a circRNA vaccine, circRNA-LNPs are taken up by cells through fusion with the endosomal membrane. Next, circRNAs are translated into proteins by ribosomes after they enter the cytosol. These antigens can trigger immune responses as follows ^[101]^: (1) The proteasome complex breaks down intracellular antigens into smaller molecules, which are then displayed on the cell surface of CD8^+^ T cells *via* attachment to MHC I (MHC I is present on the surface of almost all nucleated cells). T lymphocytes are activated and prepare to provoke a cellular immune response when they recognize and attach to epitope-bound MHC I receptors. Once activated, cytotoxic T cells excrete cytokines such as TNFα and IFN-γ, as well as cytolytic molecules, such as perforin and granzyme. Proteases can enter targeted cells through the cell membrane pores created by these cytotoxic chemicals, initiate viral protein degradation, and bolster cellular apoptosis. (2) Secreted antigens can be absorbed by antigen-presenting cells (APCs), broken down inside endosomes, and displayed on the cell surface by MHC class II proteins. When the APC is a B lymphocyte, it can develop into a plasma cell and release IgM antibodies. When a CD4^+^ T cell interacts with the APC, the CD4^+^ T cell becomes activated; facilitates the activation of phagocytes; and advocates B cell proliferation, as well as somatic hypermutation. This procedure generates antibody variants with high antigen affinities to yield highly efficient and specific immune responses. These B cells can either be retained as memory B cells or distributed in the bloodstream as plasma cells that secrete IgG antibodies. The APC can travel to the body’s lymph nodes, participate in T cell-dependent B cell maturation, and aid in the development of a more potent and persistent humoral immune response against the vaccine antigen ^[99, 102]^ (Figure 1).

## Current circRNA vaccines

### Viral infectious disease

#### CircRNA^RBD^: SARS-CoV-2 receptor-binding domain (RBD) encoding circRNA vaccine

In this study, Qu L and colleagues synthesized a circRNA vaccine that encoded SARS-CoV-2 RBD antigens by using both ribozymatic and enzymatic strategies in RNA circularization (described by Wesselhoeft *et al*.) and encapsulated the circRNAs with LNPs. circRNA-encoded RBD antigens are also functional. Mice (female BALB/c mice aged 6–8 weeks) and rhesus macaques (male, aged 2–4 years) effectively elicited sufficient neutralizing antibodies and potent specific T cell responses against SARS-CoV-2. Moreover, compared with its mRNA counterpart, this circRNA vaccine induced larger quantities of immunogens that were more durable and evoked a greater median proportion of protective antibodies against SARS-CoV-2 and their emerging variants, such as Omicron and Delta and Th1-skewed immune responses. In addition, the study proved that the delta-specific vaccine circRNA^RBD-Delta^ could provide cross-protection against all other variants, including Omicron, and could serve as an efficient booster after two doses of the initial SARS-CoV-2 vaccination. CircRNAs that encode SARS-CoV-2-specific neutralizing nanobodies, as well as hACE2 decoys, were also tested for their therapeutic potential and were effective in neutralizing SARS-CoV-2 pseudovirus. Furthermore, in vaccinated nonhuman primates, the circRNA vaccine did not cause clinical signs of sickness or exacerbated pathology, which may be proof of its safety.^[71]^

### VFLIP-X: a SARS-CoV-2 circRNA vaccine

In the present study, VFLIP-X, a circRNA vaccine targeting SARS-CoV-2, was developed to test its potential as a new-generation COVID-19 vaccine. For the *in vivo* experiments to test the validity of this vaccine, 7-week-old female BALB/C mice were used. VFLIP is a spike protein engineered using one flexible S1/S2 linker, five proline replacements in the S2 subunit, and two cysteine substitutions to induce intermolecular disulfide bond formation. VFLIP-X contains six reasonably replaced amino acids rationally chosen based on the co-mutation (D614G) discovered in all SARS-CoV-2 variants and five mutations (E484K, K417N, L452R, N501Y, and T478K) co-identified in multiple variants of concern and variants of interest. The circRNAs were produced by T7 RNA polymerase-based *in vitro* transcription and encapsulated in LNPs. According to the research, VFLIP-X imparts neutralization against SARS-CoV-2, stimulates the generation of cross-neutralizing antibodies against SARS-CoV-2 variants, and activates humoral and cellular immune responses against B. 1.1.529 variant. This study identified that a SARS-CoV-2 circRNA vaccine encoding a relatively stable VFLIP-X spike immunogen would be suitable as a next-generation COVID-19 vaccine guarding against existing and developing SARS-CoV-2 variants.^[103]^

### Tumor immune therapy

#### Cytokine-encoding circular mRNA for cancer therapy

An innovative form of circular mRNA, named cmRNA, was developed in the study. It is produced using the PIE system, which utilizes a novel spacer1 and E29 IRES to guide protein translation and is encapsulated in LNPs. This type of circular mRNA is more resilient than linear mRNA and can mediate a larger amount of protein production than linear mRNA can. Tumor cells and mouse models were used for the *in vitro* and *in vivo* experiments, respectively. The *in vitro* and *in vivo* experiments confirmed its ability to efficiently express various proteins, suggesting its use as a universal vector for protein expression. Intratumoral injection of a cmRNA compound encoding four cytokines induced a significant tumor-repressive effect. It activates immune cells, especially T cells, and collaborates with anti-PD-1 antibodies to promote total immune cell penetration into the tumor, exerting a strong antitumor effect. These results indicate that this novel type of naked cmRNA has the potential to serve as an RNA platform for the development of various intratumoral therapies.^[70]^

### CircRNA^OVA-luc^-LNP vaccine

A circRNA^OVA-luc^-LNP (OVA[257-264]-luciferase-coding circRNA) vaccine was constructed using the PIE system and encapsulated in LNPs. Three mouse tumor models were established to evaluate the efficacy of the vaccine. circRNAs demonstrate better stability and trigger longer-lasting protein expression. circRNA-LNPs elicited a potent innate immunological response and a remarkable antigen-specific response. This circRNA-LNP vaccine has shown great efficacy in suppressing the advancement of immune-exclusive tumors, inducing complete tumor regression in immunological desert tumors, and preventing cancer cell metastasis. In addition, circRNA-LNPs can collaborate with adoptive cell transfer therapy and completely repress the progression of late-stage immune-exclusive tumors by reinforcing the persistence of TCR-T cells, demonstrating the potential of RNA vaccines as tumor therapeutics.^[104]^

As shown in Table 1, the characteristics of the circRNA vaccine, including its high stability, efficacy in protein expression, stable and durable expression products, and ability to initiate immune responses, are why it is an attractive alternative to the aforementioned vaccines. Additionally, circRNA vaccines are superior to mRNA vaccines. Moreover, its manufacturing procedures and preservation conditions are simple and economical, making it more suitable for mass production and wide applications.

**Table 1:** Comparison of the emerging circRNA vaccines

Vaccine	Target immunogen	RNA circularization strategy	Delivery method	Structure to initiate translation
circRNA^RBD^	SARS-CoV-2 RBD (receptor- binding domain) antigen	Enzymatic and ribozymatic (T4 RNA (group ligase) I intron autocatalysis strategy)	LNP	CVB3 IRES
VFLIP-X	SARS-VFLIP-CoV-X 2 spike protein	Enzymatic (T4 RNA ligase)	LNP	CVB3 IRES
Cytokine-encoding circular mRNA	4 cytokines (active IL-15, IL- 12sc, GM-CSF, IFN-a 2b)	Ribozymatic (group I intron autocatalysis strategy)	LNP	E29 IRES
circRNA^OVA-luc^-LNP	OVA (257–264)-luciferase	Ribozymatic autocatalysis (group strategy) I intron	LNP	CVB3 IRES

## Conclusions

**Figure 2:**
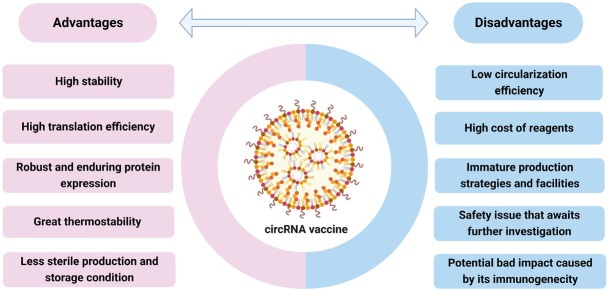
Advantages and disadvantages of circRNA vaccine.

Owing to the many advantages of circRNA, for example, high stability and achievable high translation efficiency, we used it as a vaccine. However, additional studies and improvements are required before circRNA vaccination becomes widely available. The literature has shown that circRNA vaccines do not lead to clinical signs of sickness, and further investigation is required to determine the safety of circRNA vaccines. Moreover, the impact of circRNA immunogenicity on vaccine development, which may act as an inhibitor of vaccine development, remains unclear. The classical methods described by Wesselhoeft *et al*. have been adopted and successfully used in the literature aiming to generate circRNA vaccines. However, limitations persist in the current circRNA synthesis techniques, such as low circularization productivity and the high expense of reagents such as enzymes. The stage of the current production strategies and facilities is too immature to produce massive quantities, which is an important factor restricting the application of circRNA vaccines (Figure 2). With future technological advances, we envision a broad application of circRNA vaccines in clinical trials to prevent infectious diseases and suppress tumor malignancies.
